# Emergency Department Patients Who Leave Before Treatment Is Complete

**DOI:** 10.5811/westjem.2020.11.48427

**Published:** 2021-02-26

**Authors:** Courtney M. Smalley, Stephen W. Meldon, Erin L. Simon, McKinsey R. Muir, Fernando Delgado, Baruch S. Fertel

**Affiliations:** * Emergency Services Institute, Cleveland Clinic Health System, Cleveland, Ohio; † Cleveland Clinic Lerner College of Medicine of Case Western Reserve University, Cleveland, Ohio; ‡ Akron General Medical Center, Department of Emergency Medicine, Akron, Ohio; § Northeast Ohio Medical University (NEOMED), Rootstown, Ohio; ¶ Cleveland Clinic Health System, Enterprise Quality and Patient Safety, Cleveland, Ohio

## Abstract

**Introduction:**

Emergency department (ED) patients who leave before treatment is complete (LBTC) represent medicolegal risk and lost revenue. We sought to examine LBTC return visits characteristics and potential revenue effects for a large healthcare system.

**Methods:**

This retrospective, multicenter study examined all encounters from January 1–December 31, 2019 at 18 EDs. The LBTC patients were divided into left without being seen (LWBS), defined as leaving prior to completed medical screening exam (MSE), and left subsequent to being seen (LSBS), defined as leaving after MSE was complete but before disposition. We recorded 30-day returns by facility type including median return hours, admission rate, and return to index ED. Expected realization rate and potential charges were calculated for each patient visit.

**Results:**

During the study period 626,548 ED visits occurred; 20,158 (3.2%) LBTC index encounters occurred, and 6745 (33.5%) returned within 30 days. The majority (41.7%) returned in <24 hours with 76.1% returning in 10 days and 66.4% returning to index ED. Median return time was 43.3 hours, and 23.2% were admitted. Urban community EDs had the highest 30-day return rate (37.8%, 95% confidence interval, 36.41–39.1). Patients categorized as LSBS had longer median return hours (66.0) and higher admission rates (29.8%) than the LWBS cohort. There was a net potential realization rate of $9.5 million to the healthcare system.

**Conclusion:**

In our system, LSBS patients had longer return times and higher admission rates than LWBS patients. There was significant potential financial impact for the system. Further studies should examine how healthcare systems can reduce risk and financial impacts of LBTC patients.

## INTRODUCTION

Emergency department (ED) crowding has major implications for a healthcare system. One population of patients directly affected by crowding are those who arrive to the ED for evaluation and ultimately leave before treatment is complete (LBTC). Losing these patients prior to visit completion can result in harm for the patient and missed revenue opportunities for the healthcare system. Many systems use LBTC or the vernacular left without being seen (LWBS) as a marker for ED performance. The Hospital Outpatient Quality Reporting Program through the Centers for Medicare and Medicaid Services (CMS) collects pay for quality data, which requires hospitals to submit information on certain metrics to measure patient care outcomes.^1^

“Timely and effective care-Emergency Department (ED) throughput,” OP-22, a metric that tracks LWBS, is one of many ED metrics collected to determine quality.^2^ The national average LWBS is 2%, and many hospitals strive to have an LWBS at or below the national average. The Fourth Emergency Department Benchmarking Alliance (EDBA) Summit published the most updated definitions of key language and vocabulary that should be used when defining key terminology for regulatory definitions. We have chosen to use the standardized definitions from the EDBA consensus statement published in 2020.^3^ In the most current EDBA definitions, the LBTC metric includes patients who LWBS, left against medical advice (AMA), and eloped. In their current definitions, LWBS is defined as “the proportion of patients who leave the ED before initiation of the medical screening exam (MSE).”

The EDBA additionally defines the group of AMA and eloped patients together as left subsequent to being seen (LSBS), as follows: “the proportion of patients who leave the ED after evaluation by licensed care provider qualified to complete a medical screening examination and initiation treatment but before the disposition decision by the care provider.” For simplicity in separating these two groups, we will use the terminology LWBS as patients who left without MSE completed, and LSBS as patients who had an MSE completed and ultimately eloped or left AMA.

Prior studies have characterized the LWBS population to determine the acuity of complaints, risk of missed diagnosis, return patterns, and admission rates. One recent study in a multi-hospital academic health system demonstrated that LWBS patients tended to have lower-acuity complaints and increased ED utilization.^4^ Another study in an academic pediatric ED showed that LWBS patients with higher acuity level and increased number of ED visits had high rates of admission on return visits.^5^ LWBS rates have been shown to increase during the night shift and when EDs are on diversion status.^6^ When examining return characteristics of LWBS patients, many studies have demonstrated that this patient population seeks additional medical care after leaving the ED.^7^–^9^

As a method of reducing risk, many EDs have successfully implemented programs to reduce LWBS such as creating door-to-room time goals.^10^–^12^ One study focused on Emergency Severity Index (ESI) level 2, a higher risk group of patients, and implemented a direct bedding protocol to reduce LWBS and found that odds of LWBS were lower after intervention.^13^ Another study examined optimal door-to-room times to minimize LWBS and found that times less than 20 minutes and more than 35 minutes were associated with significant differences in LBTC rates.^10^

Preventing LBTC patients from leaving is also a financial opportunity for healthcare systems. One study examined front-end practices by placing a physician in triage to study effects on LWBS and financial implications. Even with increased operating costs secondary to placing a physician in triage, the study still found a total earnings and cash flow benefit with a reduction in LWBS.^14^

**Population Health Research Capsule**

What do we already know about this issue?

*Emergency department patients who leave before treatment is complete (LBTC) represent medicolegal risk and lost revenue*.

What was the research question?

*We sought to examine LBTC return visits characteristics and potential revenue effects for a large healthcare system during 2019*.

What was the major finding of the study?

*Of the LBTC visits examined, 41.7% returned in <24 hours and 23.2% were admitted with a net potential realization rate of $9.5 million*.

How does this improve population health?

*Further studies should examine how healthcare systems can reduce the medical risk and financial impacts of this high-risk population*.

The goal of our study was to examine all patients who LBTC in a large integrated health system over a one-year period. We further defined LBTC as patients who left before MSE was complete (LWBS) and patients who left after MSE was complete but before disposition (LSBS), as per EDBA definitions. We sought to determine overall 30-day return rate within our own system, whether patients returned to the index ED where they presented on their first visit, median time to return, and admission rate. We studied factors that could contribute to differences such as ED facility type and whether patients were primarily LWBS or LSBS. Additionally, we explored the potential revenue effects on professional and technical billing fees from patients categorized as LBTC.

## METHODS

### Study Design

This was a retrospective multicenter study involving 18 EDs across a large, integrated healthcare system. This study was approved by the institutional review board of the healthcare system as a quality improvement project.

### Setting

All EDs were included in the analysis. The EDs in the healthcare system were comprised of two urban academic teaching EDs, four urban community EDs, four suburban community EDs, six free-standing EDs (FSED), and two pediatric EDs (PED), with a total annual census of 626,548 patient encounters during the time period of the study. All sites used the same electronic health record (EHR) system allowing for common accessibility and data acquisition across the system.

### Intervention and Data Collection

We included all ED visits in the analysis. Data were collected on all patients within the system who were categorized as LBTC – defined in our EHR as any patients with the following dispositions from January 1, 20191–December 31, 2019: LWBS; eloped; or AMA. To better describe the group that eloped and AMA based on using the most recent EDBA definitions, we characterized this group as LSBS. A visit was excluded if the patient had been deemed “Arrived in Error.” We collected data for the following: return rate within 30 days within the system; time elapsed from initial presentation calculated in median hours; return rate to index ED; and admission rate to the hospital.

Time elapsed from initial presentation was split into four categories: 0–23 hours; 24–47 hours; 2–10 days; and 11–30 days. Additionally, we then divided the data by facility type and whether patients were categorized as LWBS or LSBS. Additional markers collected on patient visits included whether patients returned to the index ED or to a different ED in the system, as well as admission rate to the hospital upon return. We collected data for the system as a whole, which we then examined by ED facility type. Facility types were defined as urban academic, urban community, suburban community, free-standing ED, and dedicated PED.

We collected financial data on all patients who met criteria for LWBS and LSBS populations and created a model to determine potential lost revenue. The revenue calculations were modeled as if the patient had hypothetically never left the ED. To model the financial impacts we collected patient acuity information. Acuity was based on the Emergency Severity Index (ESI) triage tool and recorded as an ESI level 1–5.^15^ The average acuity-specific charges during the study time period were also collected for each of the individual EDs in the system. Data from the EHR allowed for determination of patient insurance information, and data from the healthcare system’s administrative financial reporting system provided payor-specific contractual adjustment rates and site-specific realization rates as defined by “professional” charges, or physician fees, and “technical” charges, or facility fees. Average realization rate was defined as the insurance payment divided by total charges.

If there was no acuity recorded for an encounter, the visit was defaulted to ESI-4 and assigned fees accordingly. The encounters were defaulted to ESI-4 as we did not want to overestimate the financial impact of these encounters. All LBTC visits with either missing or “suboptimal” charges were included in the model. We defined suboptimal charges as either a professional or technical charge existing on the encounter that was less than the site-specific, acuity-specific average charge. Because insurance information could be collected as well, the average realization rate was calculated for each patient encounter during the study time period.

Two other processes were modeled to ensure the LWBS population was accounted for in the financial data as these patients did not complete an MSE. First, for patients who were charged less than the site-specific average during the study time period for their corresponding acuity level, we calculated the difference between the average and their own professional and technical fees charged. Second, once LWBS patients identified with suboptimal charges or no charges had undergone the process above, we then applied the average site-specific realization rate to professional and technical fees, as insurance carriers were recorded for all patients. We did not calculate actual realization rate but instead calculated an expected realization, based on applying actual insurance information to modeled charge details. We used this hypothetical model to project potential reimbursements. We did not include bad debt or charity care but did include co-pays into the model. We did not examine whether patients returned and, therefore, did not subtract this payment from our initial projected payment.

We conducted statistical analyses using SAS software (SAS Institute, Inc., Cary, NC). Descriptive univariate and quantile statistics were computed, with confidence intervals (CI) for the proportion of admits and returns, as well as medians and means for the other variables studied (return hours, etc). A significance level of 0.05 was used for all tests.

## RESULTS

**Figure 1:**
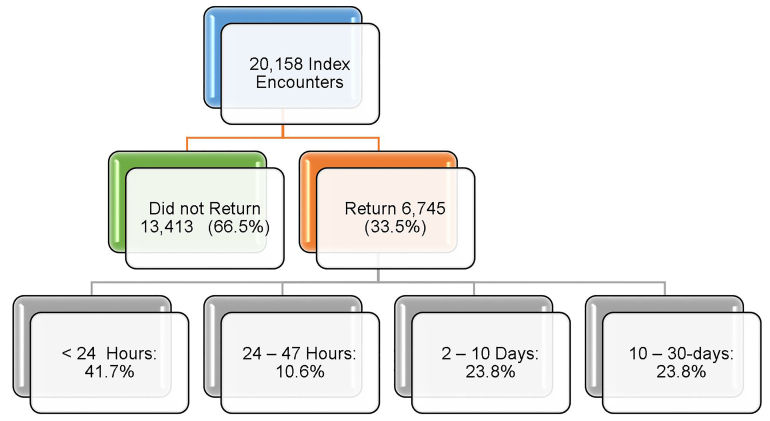
Descriptive characteristics of patients who left before treatment was completed.

During the study period, the hospital system had a total of 626,548 ED visits. There were 20,158 index encounters LBTC on this initial ED visit (3.2%). Of these index encounters, 2753 (13.7%) had no acuity recorded and 62% of this group did not return within 30 days. Within 30 days of their initial visit, 33% (6745) of these patients returned to an ED in the system. The majority of these patients (41.7%) returned in less than 24 hours; 10.6% of returns occurred within 24–47 hours; 23.8% within 2–10 days; and 23.8% within 11–30 days, (Figure 1). Overall, 5138 (76.2%) patients returned in the first 10 days of the index encounter. The median return hours for all 30-day return patients was 43.3 hours (95% CI, 41.5 – 45.3), while 66.4% (95% CI, 65.2 – 67.5) returned to the index ED with a median return of 59.8 hours (95% CI, 50.9 – 65.6) and 33.6% (95% CI, 32.5 – 34.8) returned to a different ED within the healthcare system with a median return of 20.5 hours (95% CI, 18.2 – 22.8). The admission rate for all patients categorized as LBTC was 23.2% (95% CI, 22.2–24.2) compared to the healthcare system admission rate, which was 25.4%, (Table 1).

When examining the disposition by facility type, the largest percentage of the total system 30-day returns originated from our two urban academic sites. When examining percentage of returns by index ED category, 34% (95% CI, 33.0 – 35.2) of urban academic LBTC encounters returned within 30 days, representing 37.3% of the healthcare system’s total 30-day LBTC returns. Free-standing EDs and dedicated PEDs had the lowest number of 30-day returns at 28.5% (95% CI, 26.6 – 30.4) and 21.6% (95% CI, 18.5 – 24.7), respectively, representing 8.9% and 2.2% of the system’s total 30-day returns. Urban and suburban community hospitals had 37.8% (95% CI, 36.4 – 39.1) and 32.0% (95% CI, 30.7 – 33.3), respectively, at 30 days, representing 27.8% and 23.8% of the system’s total 30-day returns.

We found a significant difference when comparing median time of return for FSED and urban community; when analyzing the difference between median return hours, the urban community and urban academic comparisons were significant at the 0.05 level. All hospital types had the highest return rate in the first 24 hours. When examining admission rates, we found that suburban community and FSEDs had the highest rates at 28.7% (95% CI, 26.5 – 30.9) and 27.1% (95% CI, 23.5 – 30.6), respectively. Pediatric EDs had the lowest admission rate at 7.33% (95% CI, 03.1 – 11.6). However, when compared to the admission rate for the dedicated PEDs in the system (10.4%) it was not significantly lower. When examining by facility type, most patients returned to the index ED with the exception of FSED patients who only returned back to the index ED 47.7% (95% CI, 43.7 – 51.7) of the time (Table 2).

When examining the differences in patients categorized as LWBS vs LSBS, we found that 35.6% (95% CI, 34.6 – 36.7) returned within 30 days with a median return hours of 23.9 (95% CI, 21.9 – 27.9), and admission rate of 14.5% (95% CI, 13.3 – 15.8), compared to 32.0% (95% CI, 31.1 – 32.8) of LSBS who returned within 30 days with a median return hours of 66.0 (95% CI, 59.3 – 68.7) and admission rate was 29.8% (95% CI, 28.4 – 31.3). In both categories, the percentage who returned to the index ED was 64.0% (95% CI, 62.3 – 65.7) and 68.2% (95% CI, 66.7 – 69.7), respectively (Table 2). When comparing the differences between patients categorized as LWBS to LSBS, there were no significant differences that indicated a particular type of ED facility had effects on 30-day return encounters, hours between visits, admission rate, or return to index ED. Overall results for time elapsed since index ED visit, LBTC categorization, and admissions rates are shown in Table 3.

Across the system, the potential net revenue annualized from LWBS and LSBS approximated 9.5 million dollars (Table 4). The annualized potential net professional revenue was two million dollars, and the potential net technical revenue equaled 7.5 million dollars. When comparing facility type, urban academic EDs had the most potential revenue for professional and technical fees (Table 4). When comparing disposition category by examining the potential net revenue from patients who left from the ED waiting room before MSE was complete (LWBS) vs patients who left after MSE was complete either from the waiting room or from the ED (LSBS), LWBS patients amounted to a significant unrealized potential net professional and technical charge of 5.6 million dollars (Table 5).

## DISCUSSION

Patients who leave the ED prior to completion of their visit represent potential medicolegal risk as well as lost revenue for the healthcare system. Examining when these patients leave during the course of the emergency visit is important so that hospital systems can create initiatives to ensure that patients complete their visits and receive emergency care. Our study was unique in that we attempted to characterize not only time-to-return hours and admission rate, but we also examined factors that may play a role in return practices of these patients. To achieve this goal and determine whether there were significant differences, we examined all LWBS patients and LSBS patients. Our study found that patients who left after being roomed in the ED had a longer median hour to return albeit with significantly higher admission rates. There seemed to be no difference between the two groups when examining the percentage return rate, return to the index ED, or facility type to which they initially presented.

To our knowledge, this is the first study to examine and differentiate the entire population of patients that leaves the ED. Previous studies have examined LWBS patients in an effort to determine medical complexity and risk to the healthcare system but only examined the group that leaves before being seen by a physician. Li et al found that this group of patients likely had lower acuity chief complaints and higher return rates, with an overall lower admission rate.^3^ While previously lumped together in this broad category, this group is not homogeneous. Our study also included the eloped and AMA population, as well, to clearly delineate the characteristic differences between these two patient groups who left from the waiting room before MSE was complete and those patients who left the ED after MSE was complete.

This data gives the healthcare system a better representation of true risk between these two patient groups. It helps characterize whether there are specific differences among these populations and whether targeted interventions could be applied for each population. One could reason that LSBS patients may have higher acuity issues, as they stayed long enough to finish the MSE and potentially be seen in the main ED in the first place. Additionally, once the population of LSBS left, this group may have had more of a workup and been given some insight into return precautions, causing longer median return times for their second visit.

Another unique aspect of our study was its examination of ED facility type to determine whether significant differences existed among the patient groups. We found that only FSEDs and PEDs had significantly lower rates of return. Additionally, FSEDs had fewer patients return back to the index ED when compared to other facility types. It is possible that this group of patients may have re-presented to an alternative ED in the system that had inpatient capabilities with the thought that they might need to be admitted. All facilities had the majority of their returns in the first 24 hours. Admission rates were similar across different ED facility types with the exception of the dedicated PEDs, which had comparable admission rates to their lower typical specialty-population admission rate. Future studies should examine how each of these LBTC patient groups individually present at certain types of EDs to determine whether targeted interventions by ED type could facilitate a drop in LBTC numbers.

When we examined the LBTC group as a whole, our study found many of these patients returned to the same ED within 24 hours and had admission rates similar to our typical hospital admission rates (23.2% vs 25.4%). A prior study examined admission rates for this vulnerable population and found lower admission rates (11.5%) than their overall ED average, likely based on the findings that these patients more frequently presented with lower acuity complaints.^3^ However, when further categorized into LWBS and LSBS, admission rates differed. Importantly, despite generally lower acuity, patients categorized as LWBS had an admission rate of 14.5% on return visit. Differentiating the LWBS from the LSBS population might allow more directed or targeted interventions for these groups, recognizing that patients who leave from the waiting room prior to MSE have overall lower admission rates and quicker return rates when compared to the LSBS patient population, which includes those patients who elope or leave AMA.

When attempting to quantify unrealized revenue effects, our study demonstrated that the LWBS population has significant potential financial impact on the healthcare system. Overall, LWBS patients have more opportunity loss than LSBS patients, as many of these patients do not stay long enough into their visit to incur the professional and technical charges that would be incurred if they had completed a full visit. Additionally, many of the AMA patients received full charges for their visits. This population of AMA patients likely does not provide significant additional revenue opportunities.

Healthcare systems should consider initiatives aimed at keeping patients within their own system to improve market share and increase overall ED revenue (and potential hospital revenue resulting from subsequent admissions) by addressing the issue of LWBS patients who leave the waiting room early in their visit prior to MSE and addressing processes that influence LWBS decisions. Ultimately, we created this hypothetical financial model to better understand the costs for additional resources that would be needed (nursing, physician extenders, physicians, etc) to fund initiatives to reduce LBTC. Further studies should examine opportunity cost for developing programs that reduce LWBS and LSBS to improve patient safety outcomes and reduce financial losses.

## LIMITATIONS

In this study we were unable to account for patients who may have re-presented to EDs outside of our healthcare system. While patients may have returned to other hospitals outside of our system, our healthcare system does have multiple hospitals throughout the area and holds a large percentage of the market share. Second, we were unable to account for inter-rater reliability for ESI triage levels at different EDs within the system in that patients could have been mis-triaged or potentially up/downgraded in triage. As different staff at our facilities are triaging patients at each hospital, it could account for differences in ESI acuity levels on re-presentation. Our healthcare system used the EDBA definitions and our classifications of disposition were determined by our frontline waiting room staff. Nursing was educated on these definitions, but we cannot exclude that some patients may have been mischaracterized.

Further, because we did not examine the subtype population of patients with high-frequency ED utilization, we were not able to account for whether patients who returned were having different chief complaints from their initial presenting complaint prior to LBTC. Additionally, when comparing LWBS and LSBS patients, we were not able to adjust for ED type or ESI level. While we attempted to study the entire LBTC group as a whole, we also acknowledge that each group has different characteristics and further examination of each subtype may better help create projects that reduce leaving from the ED before the visit is complete. Lastly, since we were looking at markers for the healthcare system as a whole to make recommendations for overall system improvement, some sites may have characteristics that differ from our primary findings.

Regarding financial data limitations there is no ideal method to calculate realization rates per encounter. We attempted to account for this revenue stream by defaulting any encounters without an ESI acuity level to ESI level 4. These triage complaints could have been ultimately higher or lower acuity level. Additionally, for suboptimal charges, we had to take the average site encounter charge for particular ESI levels and calculate the difference between the billed charge and the average site-encounter charge. However, because we were able to gather the insurance information for all of these patients, we were able to get a net realization charge for potential lost revenue.

Another major limitation of our financial model is that we did not account for return after leaving the ED, ie, this initial financial calculation was only meant to demonstrate the potential income stream lost by patients who leave the ED. Further analysis would have to account for patients who subsequently return and create a financial model to adjust for re-captured revenue. Our study demonstrated that more financial opportunity was available for patients categorized as LWBS before MSE was completed and that decreasing the rate of leaving would increase financial opportunities for the healthcare system.

## CONCLUSION

In our multicenter study, patients who left AMA or eloped (LSBS) had longer time to return and much higher admission rates with resultant less financial loss to the healthcare system than patients who left without being seen before a medical screening exam was completed. Facility type had less influence on these factors. Further studies should examine how healthcare systems can reduce the prevalence of patients who leave before treatment is completed since this group of patients represents an area of lost revenue for the healthcare system.

## Figures and Tables

**Table 1:** Left before treatment complete (return encounter characteristics for the healthcare system and based on hospital type).

	System total	Urban academic	Urban community	Suburban community	FSED	PEDs
LBTC encounters N	20,158 (3.2%)	7,364 (5.9%)	4,966 (3.1%)	5,019 (2.7%)	2,114 (1.9%)	695 (1.5%)
Overall 30-day LBTC returns N (%)	6,745 (33.5%)	2,513 (34.1%)	1,875 (37.8%)	1,605 (32.0%)	602 (28.5%)	150 (21.6%)
Median return (Hours)	43.1	41.0	53.7	40.0	37.2	33.2
30 day admit rate N (%)	1,565 (23.2%)	565 (22.5%)	365 (19.5%)	461 (28.7%)	163 (27.1%)	11 (7.3%)
30-day returns to index ED N (%)	4,476 (66.4%)	1,626 (64.7%)	1,382 (73.7%)	1,082 (67.4%)	287 (47.7%)	99 (66.0%)

*LBTC*, left before treatment complete; *FSED*, free-standing emergency department; *PED*, pediatrics; *ED*, emergency department.

**Table 2:** Comparing patients who left without being seen vs patients who left subsequent to being seen.

	LWBS	LSBS	System total
LBTC encounters N	8,206	11,952	20,158
Overall 30-day LBTC returns N (%)	2,924 (35.6%)	3,821 (32.0%)	6,745 (33.5%)
Median return (Hours)	23.7	66.0	43.1
30-day admit rate N (%)	425 (14.5%)	1,140 (29.8%)	1,565 (23.2%)
30-day returns to index ED N (%)	1,871 (64.0%)	2,605 (68.2%)	4,476 (66.4%)

*LWBS*, left without being seen; *LSBS*, left subsequent to being seen; *LBTC*, left before treatment complete; *ED*, emergency department.

**Table 3:** Comparing dispositions when patients left without being seen vs. who left subsequent to being seen.

	0 – 23 Hours	24 – 48 Hours	2 – 10 Days	10 – 30 days	Total 30-day returns
Index ED disposition: all left before treatment complete					
Overall					
Return encounters	2,815	716	1,607	1,607	6,745
Return encounters: admitted	680	153	368	364	1,565
% Admitted (of total returns)	24.2%	21.4%	22.9%	22.7%	23.2%
Hours between visits (average)	7.7	37.1	125.0	453.7	144.9
Hours between visits (median)	5.2	38.5	116.1	445.6	43.3
Index ED disposition: LWBS					
Overall					
Return encounters	1,465	286	601	572	2,924
Return encounters: admitted	216	36	80	93	425
% Admitted (of total returns)	14.7%	12.6%	13.3%	16.3%	14.5%
Hours between visits (average)	6.7	36.8	126.2	455.4	121.8
Hours between visits (median)	2.8	37.9	116.9	452.4	23.9
Index ED disposition: LSBS					
Overall					
Return encounters	1,350	430	1,006	1,035	3,821
Return encounters: admitted	464	117	288	271	1,140
% Admitted (of total returns)	34.4%	27.2%	28.6%	26.2%	29.8%
Hours between visits (average)	8.9	37.3	124.2	452.8	162.7
Hours between visits (median)	7.3	38.8	115.6	441.8	66.0

*LWBS*, left without being seen; *LSBS*, left subsequent to being seen; *LBTC*, left before treatment complete; *ED*, emergency department.

**Table 4:** Overall system and emergency department facility type comparison of potential professional and technical fees for patient who left before treatment was completed.

ED category	Potential professional fees					

	Encounters missing charges	Charges	Avg. additional charge/encounter	Avg. realization rate	Net	Net per encounter
System overall	16,723	$8,332,286	$498	24.2%	$2,020,273	$121
Urban academic	6,591	$2,691,244	$408	29.2%	$785,575	$119
Urban community	3,785	$1,987,387	$525	17.3%	$343,604	$91
Suburban community	4,203	$2,820,195	$671	20.8%	$587,756	$140
FSED	1,514	$491,224	$324	31.5%	$154,683	$102
PED	630	$342,237	$543	19.6%	$67,201	$107
	
	Potential technical fees					
	
System overall	17,749	$29,171,876	$1,644	25.9%	$7,547,629	$425
Urban academic	6,893	$13,118,331	$1,903	26.7%	$3,497,287	$507
Urban community	4,134	$5,693,974	$1,377	21.0%	$1,193,706	$289
Suburban community	4,419	$7,463,106	$1,689	28.2%	$2,101,090	$475
FSED	1,644	$2,116,398	$1,287	28.6%	$604,826	$368
PED	659	$780,066	$1,184	26.4%	$205,939	$313

*ED*, emergency department; *LBTC*, left before treatment complete; *Avg*, average; *FSED*, free-standing emergency department; *PED*, pediatric emergency department.

**Table 5:** Comparison of potential professional and technical fees for left before treatment complete patients.

Dispo category	Potential professional fees						

	Original $0 charges	Original charges < average*	Charges	Avg. additional charge/encounter	Avg. realization rate	Net	Net per encounter
System overall	12,048	4,675	$8,332,286	$498	24.2%	$2,020,273	$121
LWBS*	8,175	24	$5,501,374	$671	21.7%	$1,193,498	$146
LSBS**	3,873	4,651	$2,830,913	$332	26.7%	$755,876	$89
	
	Potential technical fees						
	
System overall	8,357	9,392	$29,171,876	$1,644	25.9%	$7,547,629	$425
LWBS*	7,610	585	$16,108,900	$1,966	27.4%	$4,413,713	$539
LSBS**	747	8,807	$13,062,975	$1,367	24.6%	$3,208,764	$336

* LWBS (left without being seen): patients who leave the ED before initiation of medical screening examination.

** LSBS (left subsequent to being seen): patients who leave the ED after evaluation by licensed care provider qualified to complete a medical screening examination and initiate treatment but before the disposition decision by the care provider.

*Avg*, average.
